# An Update on Pentacyclic Triterpenoids Ursolic and Oleanolic Acids and Related Derivatives as Anticancer Candidates

**DOI:** 10.3390/antiox13080952

**Published:** 2024-08-06

**Authors:** Diana Similie, Daliana Minda, Larisa Bora, Vladislavs Kroškins, Jevgeņija Lugiņina, Māris Turks, Cristina Adriana Dehelean, Corina Danciu

**Affiliations:** 1 Department of Pharmacognosy, Faculty of Pharmacy, “Victor Babeș” University of Medicine and Pharmacy, Eftimie Murgu Square, No. 2, 300041 Timisoara, Romania; diana.similie@umft.ro (D.S.); larisa.bora@umft.ro (L.B.); corina.danciu@umft.ro (C.D.); 2 Research and Processing Center of Medicinal and Aromatic Plants, “Victor Babeș” University of Medicine and Pharmacy, Eftimie Murgu Square, No. 2, 300041 Timisoara, Romania; cadehelean@umft.ro; 3 Institute of Chemistry and Chemical Technology, Faculty of Natural Sciences and Technology, Riga Technical University, Paula Valdena Str. 3, LV-1048 Riga, Latvia; vladislavs.kroskins@rtu.lv (V.K.); jevgenija.luginina@rtu.lv (J.L.); maris.turks@rtu.lv (M.T.); 4 Department of Toxicology, Drug Industry, Management and Legislation, Faculty of Pharmacy, “Victor Babeș” University of Medicine and Pharmacy Timișoara, Eftimie Murgu Square, No. 2, 300041 Timisoara, Romania; 5 Research Center for Pharmaco-Toxicological Evaluation, “Victor Babeș” University of Medicine and Pharmacy, Eftimie Murgu Square, No. 2, 300041 Timisoara, Romania

**Keywords:** ursolic acid, oleanolic acid, pentacyclic triterpenoids, cancer, antiproliferative, cytotoxic, proapoptotic, chemopreventive, in vitro, in vivo, clinical trials

## Abstract

Cancer is a global health problem, with the incidence rate estimated to reach 40% of the population by 2030. Although there are currently several therapeutic methods, none of them guarantee complete healing. Plant-derived natural products show high therapeutic potential in the management of various types of cancer, with some of them already being used in current practice. Among different classes of phytocompounds, pentacyclic triterpenoids have been in the spotlight of research on this topic. Ursolic acid (UA) and its structural isomer, oleanolic acid (OA), represent compounds intensively studied and tested in vitro and in vivo for their anticancer and chemopreventive properties. Since natural compounds can rarely be used in practice as such due to their characteristic physico-chemical properties, to tackle this problem, their derivatization has been attempted, obtaining compounds with improved solubility, absorption, stability, effectiveness, and reduced toxicity. This review presents various UA and OA derivatives that have been synthesized and evaluated in recent studies for their anticancer potential. It can be observed that the most frequent structural transformations were carried out at the C-3, C-28, or both positions simultaneously. It has been demonstrated that conjugation with heterocycles or cinnamic acid, derivatization as hydrazide, or transforming OH groups into esters or amides increases anticancer efficacy.

## 1. Introduction

Despite scientific progress and the development of numerous treatment methods, cancer is the second cause of death in the world after cardiovascular diseases [1]. According to Global Cancer Statistics, every year, approximately 19 million people are diagnosed with cancer worldwide [2]. In 2020, there were more than 10 million deaths caused by cancer in the global population [2,3]. It is estimated that by 2030, the cancer incidence rate will increase by up to 40% [4]. This disease manifests physical, emotional, and financial pressure on the patients and also on the national health systems. Patients from underdeveloped or developing countries have low survival rates due to late detection and inaccessibility to treatment [1]. Current cancer treatment includes surgical treatment, radiotherapy, chemotherapy, immunotherapy, hormonal therapy, and adjuvant therapy. However, none of the therapy options is ideal. They cause numerous side effects, such as immunosuppression, central nervous system (CNS) disorders, and vomiting, and do not guarantee complete healing. The difficulty of treating and eradicating this disease comes from the ability of cancer cells to develop resistance to treatment through different mechanisms, some still unexplained, but also from the limitation of drug doses due to the toxic reactions. Therefore, research into cancer treatment remains a highly interesting topic in the field.

Phytotherapy is a branch of medicine based on the use of plant products and extracts obtained from medicinal herbs in order to prevent or treat acute or chronic diseases. Worldwide, more than 8000 plants are used for therapeutic purposes [5]. In the last 40 years, natural bioactive compounds have gained an important place in the prevention and treatment of cancer. The data show that over 70% of all FDA-approved drugs are based on natural products or their derivatives, and also over 70% of the anticancer drugs on the market were developed starting from natural compounds [1,6]. Between 1981 and 2019, about a quarter of newly approved anticancer drugs were related to natural products [7]. Various secondary plant metabolites, including terpenes, terpenoids, polyphenols, and alkaloids, possess cytotoxic and chemopreventive properties [8,9]. Moreover, certain triterpenoids, flavonoids, alkaloids, and curcumins have been studied to defeat multi-drug resistance [6].

Natural products play an extremely important role in the discovery and development of new anticancer compounds with superior pharmacokinetic, pharmacodynamic, and pharmaco-toxicological properties. Some anticancer compounds of plant origin, such as *Catharanthus* alkaloids (vincristine, vinblastine), colchicine, etoposide, podophyllotoxin, topotecan, irinotecan, docetaxel, and paclitaxel, are already widely used in the treatment of various types of cancer [8,10,11]. However, the use of anticancer compounds of plant origin also presents certain limitations, including low bioavailability, rapid elimination, lack of selectivity, and restriction in metastasis [10].

Pentacyclic triterpenoids (PTs) represent a class of widespread natural compounds (over 20,000 isolated compounds) with significant therapeutic value [12]. PTs are chemical compounds with 30 carbon atoms, which can be isolated from plants, fungi, or animals [5,13,14]. They are secondary metabolites present in the plant kingdom and, as such, can be extracted mainly from roots, stem bark, leaves, or fruits [15]. They possess anti-inflammatory, antioxidant, analgesic, antidiabetic, immunomodulatory, hypolipidemic, neuroprotective, antibacterial, antiviral, and antifungal activities, becoming a topic of interest for researchers [14,15,16,17,18,19]. It is already known that PTs can be used in the treatment of various types of cancer without showing evident toxicity on healthy cells [20]. Their important therapeutic potential, increased selectivity, and efficacy make them attractive to the scientific community for the development of new cancer treatments.

**Figure 1:**
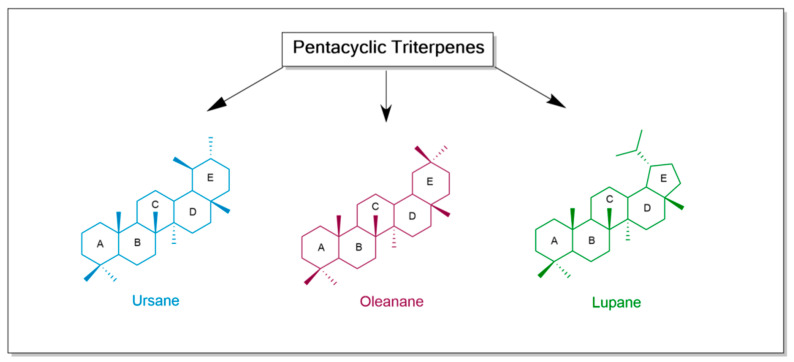
Classification of the main pentacyclic triterpenes skeletons. The structures were designed with ChemDraw 23.0.1.

Triterpenoids are oxygen derivatives of triterpenes. Pentacyclic triterpenes can be divided into the following three main classes: ursane, oleanane, and lupane type [18,21] (Figure 1). Betulinic acid (lupane scaffold), ursolic acid (ursane scaffold), and oleanolic acid (oleanane scaffold), which are valuable compounds in therapeutics, belong to the class of pentacyclic triterpenoids.

**Figure 2:**
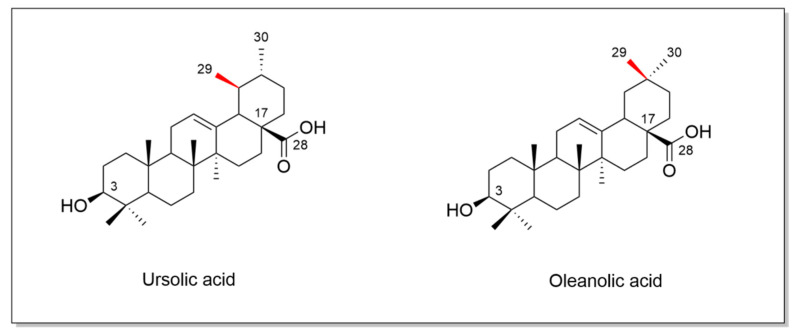
Chemical structures of UA and OA. The structures were designed with ChemDraw 23.0.1.

UA and its structural isomer, OA (Figure 2), can be extracted from various fruits, vegetables, and medicinal plants (e.g., apples, cranberries, blueberries, basil, olive, oregano, lavender) [1,4,13,22]. They are often found in nature together, in the form of free acid or as an aglycone of triterpenoid saponins [23]. When comparing UA and OA, the only difference between the chemical structures of the two compounds is the methyl group placement on the ring E.

UA and OA have been described in the literature for their multiple health benefits, having both prophylactic and curative roles [12,23,24]. The overproduction of reactive oxygen species (ROS) is strongly associated with an increased risk of cancer and chronic disease development. In this regard, antioxidants play a significant role in protecting, repairing, and mitigating the damage produced by oxidative stress. UA and OA antioxidant action is mainly related to their capacity to scavenge ROS and counterbalance the pro-oxidants/antioxidants ratio.

These two triterpenic acids have been successfully tested on various human or murine cancerous cell lines (leukemia, prostate, colon, breast, pancreatic, urinary bladder, lung, endometrial, ovarian, melanoma, hepatocellular cancer) [14,17,25,26,27]. In vitro evaluations corroborated with in vivo studies have demonstrated that the anticancer effect of these compounds can be assigned to several mechanisms. The main mechanisms included inhibition of cell proliferation, induction of apoptosis, inhibition of tumor invasion, and sensitization of cancer cells to chemotherapeutic agents [15,17,22,24,28]. UA and its derivatives are under study or have already undergone clinical studies aimed at establishing the toxicity and pharmacokinetic profile [13,29]. It was also pointed out that UA could be useful both in the prevention and treatment of cancer, including the prevention of metastases [13,30].

Although natural compounds have recognizable therapeutic potential, they can rarely be used directly in clinical practice, requiring certain structural modifications to improve bioavailability and facilitate administration [31]. Derivatization is the chemical modification of the parent structure, leading to semi-synthetic derivatives that can improve the pharmacological profile of various natural compounds. Structural modifications are used to obtain compounds with improved polarity, solubility, stability, and even pharmacodynamic action and reduced toxicity [32,33].

The limitations of UA and OA include their availability at the target site, poor solubility and bioavailability, fast metabolism, and the toxic potential of the solvents used (dimethylsulfoxide (DMSO) or dimethylformamide (DMF)) for the solubilization of these compounds [1,4,34,35]. The bioavailability of UA in plasma is limited to 500 nM [1]. These disadvantages assigned to UA caused it to be classified in the IVth class of the Biopharmaceutical Classification System [4]. To overcome these barriers, research is directed toward synthesizing UA and OA analogs with superior pharmacokinetic properties. The inclusion of these compounds in targeted-release pharmaceutical forms may also be sought. Using chemical or microbial methods, numerous researches have been directed toward obtaining ursolic and oleanolic acid derivatives with superior bioavailability [36]. One of the current methods is pharmacophore hybridization, which involves joining two compounds known to be effective through covalent bonds and obtaining a hybrid compound with superior pharmacological and toxicological properties [16].

This review aims to present the results of recent studies on the structural modification and biological evaluation of both UA and OA.

## 2. Ursolic Acid

Ursolic acid has the basic ursane skeleton. UA (3β-hydroxy-12-urs-12-en-28-oic acid) contains the following functional groups for potential chemical modifications: C17-COOH, C3-OH, and an alkene at C12-C13.

UA is one of the most abundant and studied pentacyclic triterpenoids, being present in most edible plant products (e.g., fruit cuticular wax, edible leaves, bark, flowers of medicinal plants), especially in plants from the *Lamiaceae* family [1,17]. For the first time, it was extracted from apple waxes [4]. Among the main sources for UA extraction can be listed as follows: *Mimusops caffra* E. Mey, *Ilex paraguariensis* A.St.-Hil., *Glechoma hederacea* L., *Ligustrum lucidum* L., *Centella asiatica* L., *Lysimachia clethroides* Duby., *Rosmarinus officinalis* L. (3.0%), *Salvia officinalis* L. (1.8%), *Arctostaphylos uva-ursi* L., *Vaccinium macrocarpon* Ait., *Ocimum sanctum* L., and *Eugenia jambolana* L. [12,13,17,20,37,38]. UA extraction is based both on traditional methods (Soxhlet and reflux extraction) and also on modern methods such as supercritical fluid extraction, ultrasonic, and microwave extraction [36].

Products containing UA have been widely used as an anticancer agent in traditional Chinese medicine. In the last 20 years, it has been intensively studied as a preventive and curative agent for various types of cancer due to its proven properties in in vitro and in vivo studies and even in clinical trials [13,29]. UA presents a significantly high potential due to its property of being relatively non-toxic against healthy cells, demonstrating antiproliferative activity against malignant cells [1,34]. Its anticancer action is due to several mechanisms, including antitumorigenic, antiangiogenic, and tumor growth prevention, especially in the case of breast, cervical, and colorectal cancer [16,37,38]. In vitro studies have shown that UA interferes with other molecules involved in cell signaling pathways [1]. More specifically, UA can modulate various molecular targets, including growth factors (EGF, HGF), enzymes (ATPase, COX-2, telomerase), receptors (HER-2, EAR, EGFR), pro-inflammatory cytokines (interleukins 1, 6 and 8), as well as transcription factors (STAT3, NF-κB) [39]. Furthermore, UA showed the ability to suppress NF-κB activation caused by various carcinogens (cigarette smoke, tumour necrosis factor alpha (TNFα), or H_2_O_2_) [40]. On multiple myeloma cell lines, UA has been reported to be involved in inhibiting both constitutive and IL-6 inducible STAT3 activation [41]. It was also observed that UA can cause colorectal cancer cell apoptosis by inhibiting constitutive NF-κB activation and downregulating cell survival proteins (such as Bcl-xL, Bcl-2), as well as metastatic proteins (such as MMP-9 and VEGF) [42]. Along with glycyrrhetinic acid, UA is a potential moderator of multiple resistance and a chemosensitizer [43]. Moreover, it was demonstrated that UA can radiosensitize various cancer cell lines (DU145, CT26, and B16F10) [13].

In addition to the anticancer effect, UA and its derivatives possess a wide range of pharmacological effects, including antidiabetic, antiosteoporotic, hypocholesterolemic, hepatoprotective, neuroprotective, antiviral, anti-inflammatory, and antifungal, with very low toxicity [1,17,20,22,43,44].

Antioxidant compounds intervene in the prevention of the harmful effects of ROS and the maintenance of an equilibrium between pro-oxidants and antioxidants in the cells [45]. The overproduction of ROS during oxidative stress is responsible for a series of proteins and DNA degradations, as well as structural and functional changes in cells, which could lead to genetic mutations, metabolic pathway disruptions, pro-oncogenic signaling, and, ultimately, tumor initiation [46]. Therefore, the improvement of the biological antioxidant defense system could represent a strategy to prevent carcinogenesis [47]. In this regard, the antioxidant potential of UA and OA was further evaluated.

Srinivasan and colleagues employed five different assays in order to evaluate the radical scavenging potential of various UA concentrations (20, 40, 60, 80, and 100 μg/mL). The best antiradical activity was obtained in the ferric reducing antioxidant power (FRAP) method, followed by hydroxyl radical, superoxide radical, nitric oxide radical, and 2,2-diphenyl-1-picrylhydrazyl (DPPH) radical. Noteworthily, IC_50_ value of UA in superoxide radical scavenging assay (IC_50_ = 43.35 μg/mL) was lower than those of ascorbic acid and butylated hydroxyanisole used as antioxidant controls (IC_50_ = 67.03 μg/mL and IC_50_ = 90.84 μg/mL, respectively) [25]. In the same line, Yin and Chan evaluated the antioxidant effect of 5 μM and 10 μM UA and OA. The tested compounds exerted a scavenging effect of superoxide anion (OA—50.5% and UA—33.5% at 10 μM), chelating effect on ferrous ions (OA—21.3% and UA—34.2% at 10 μM), and an inhibitory effect of xanthine oxidase activity (OA—48.6% and UA—37.4% at 10 μM). Further, the researchers observed a better antioxidant effect of UA and OA against 2,2′-azobis-(2-amidinopropane) dihydrochloride than that against 2,2′-azobis-(2,4-dimethylvaleronitrile). Moreover, OA showed a greater antioxidant effect than UA and α-tocopherol at 75 °C and 100 °C, while the effect of UA and OA was stronger than that of α-tocopherol at pH 2 and 4 [48]. High scavenging activity of hydroxyl radical was observed by Samsonowicz et al. for UA. In the tested concentration range of 0.003 to 0.016 M, UA decreased around 60% of the initial concentration of hydroxyl radicals in a concentration-dependent manner [49]. Do Nascimento et al. investigated the antioxidant effect of UA (isolated from the ethanolic extract of *Sambucus australis* Cham. and Schltdl. aerial parts) and two other derivatives employing DPPH assay. As expected, UA showed significant antioxidant activity, as well as 3β-acetoxy urs-12-en-28-oic acid, the acetylated compound. On the contrary, the formylated compound (3β-formiloxy-urs-12-en-28-oic acid) was inactive in terms of antioxidant properties [50].

Several research studies show that UA plays an important role in maintaining the intracellular redox balance by modulating oxidative stress-related indicators. A neuroprotective effect through oxidative stress modulation of UA and OA on PC12 rat adrenal gland pheochromocytoma cells was revealed by Tsai and Yin. Treatment with H_2_O_2_ or 1-methyl-4-phenylpyridinium (MPP^+^) substantially increased malonyldialdehyde (MDA) levels and decreased superoxide dismutase (SOD), catalase (CAT), and glutathione peroxidase (GPx) activities, respectively glutathione (GSH) levels. Pretreatment with UA or OA, in doses of 20 μM and 40 μM, reversed the imbalance produced H_2_O_2_ or MPP^+^ in terms of oxidative stress-related indicators. MDA formation decreased, while GSH content and antioxidant enzyme activities were preserved [51]. UA showed antioxidant properties by modulating the oxidative stress-related indicators in an experimental ICR mice liver fibrosis model induced by carbon tetrachloride. A dose-dependent inhibition of ROS and thiobarbituric acid reactive substances (TBARSs) was observed after treatment with 25 mg/kg and 50 mg/kg UA. At the same time, the activity of the antioxidant enzymes CAT, SOD, and GPx increased following treatment with UA [52]. Ramachandran and Prasad demonstrated the protective effect of 10 μg/mL UA pretreatment against UVB-induced damage in human lymphocytes. Firstly, the researchers evaluated the scavenging ability of UA on hydroxyl radicals, superoxide anion, nitric oxide, ABTS, and DPPH, the most effective scavenging effect being observed against DPPH radical (IC_50_ = 5.93 μg/mL). Further, treatment with UA 30 min before UVB exposure protected against lipid peroxidation, oxidative stress, and DNA damage by decreasing TBARS, lipid hydroperoxides, % tail DNA, and tail moment. In addition, cell viability also increased following pretreatment with UA [53]. The same group study proved that pretreatment with UA in UVB-irradiated lymphocytes increased the activity of antioxidant enzymes (SOD, CAT, and GPx) [54]. Briefly, UA exhibits potent and effective antioxidant activity by reducing oxidative stress and DNA damage, with possible additional implications in both preventive and curative cancer management.

Therefore, UA can represent a starting point for obtaining new potent compounds with antioxidant and antiproliferative effects [25]. Being a hydrophobic compound, UA requires structural chemical changes before being administered to increase its bioavailability [4,55].

### 2.1. Structural Modifications

Many research efforts have been directed towards semi-synthetic derivatives of UA with the aim of increasing the bioavailability and enhancing the anticancer effect [4]. Table 1 presents UA derivatives with proven antiproliferative effects in various in vitro and in vivo studies. The structures were designed with ChemDraw 23.0.1.

It was demonstrated that C3-OH and C17-COOH groups are extremely important for the cytotoxic activity of UA and its derivatives. Starting from this observation, maintenance or modification of one or both of these chemical groups was tried, with modifications in other positions being called miscellaneous modifications [4]. However, some studies have shown that converting the C3-OH to an acetyl group can be beneficial [1].

Plentiful attempts to improve the cytotoxic activity of UA through changes at the C3 and C28 positions are described in the literature. Numerous compounds from the class of esters, amides, and oxadiazole quinolines were obtained [20]. Among the structural changes that increase the antitumor activity and cytoselectivity are the introduction of a piperazine, homopiperazine, triazole, or guanidine moiety [1,22]. It was observed that the length of the chain of carbon atoms influences the anticancer activity, with the compounds with unbranched chains with two or three carbon atoms showing optimal activity against the MCF-7 and THP-1 cancer lines tested. Moreover, it was shown that 4-fluorobenzyl and piperazine moieties are necessary to manifest the anticancer effect [22]. It was also pointed out that 3-oxo-UA-triazolyl derivatives with o-bromo, o-chloro, or o-methoxy substitution on the aromatic ring inhibit the proliferation of MCF-7 and THP-1 cancer cell lines [22,65].

Several other studies have considered the implementation of other structural changes. It has been shown that modification of the UA structure by the introduction of thiazole on A ring and triazole or tetrazole moiety on C-28 causes a slight increase in antitumor activity [22]. Also, the introduction of various substituted benzene rings at the C-2 position and retention of the carboxyl group at C-28 proved to be beneficial against the HCT-116 cell line [66].

### 2.2. In Vitro, In Vivo, and Clinical Trials

Several research teams have attempted to identify and elucidate the antiproliferative and cytotoxic mechanisms of UA and its derivatives. Numerous in vitro studies performed using various cancer cell lines showed promising results (Table 2).

Some of the most representative studies on the anticancer properties and mechanism of UA are described below.

Young Kang et al. showed that UA has a dose-dependent antiproliferative effect on two non-small cell lung cancer cell lines A549 and H460. Also, its antiangiogenic effect was highlighted on human umbilical vein endothelial cells (HUVECs) [67]. Using four breast cancer cell lines (MCF-7, MDA-MB-231, 4T1, and HBL-100), Wang et al. demonstrated that UA inhibits malignant cell proliferation due to S-phase cell cycle arrest and induction of cell apoptosis mediated by attenuation of Bcl-2 protein, increase in Bcl-2 associated X-protein (BAX), and cleaved poly (ADP-ribose) polymerase (PARP) [68]. Furthermore, using the MCF-7 breast cancer cell line, Guo and his colleagues observed that the antiproliferative effect of UA is based on the modulation of IKK/NF-κB and RAF/ERK pathways and also on the downregulation of the phosphorylation level of PLK1 [69]. Furthermore, Lin et al. revealed that the antiproliferative effect of UA against human ovarian cancer cell line SKOV-3 is due to the intervention in the process of apoptosis, regulation of ROS, and matrix metalloproteinase (MMP), as well as downregulation of the PI3K/AKT pathway [28]. Following the administration of UA on Ca922 and SCC2095 oral cancer cells, Lin and his collaborators observed that caspase-dependent cell apoptosis was induced, and Akt/mTOR/NF-κB signaling pathways were downregulated. Also, UA determined the inhibition of the angiogenesis process in Ca922 cells and induced autophagy in OSCC cells [70].

Regarding UA derivatives, several in vitro studies proved their significant antiproliferative effect, emphasizing that certain structural modulations led to improved therapeutic efficiency. The conclusions of some of them are stated below.

**Figure 3:**
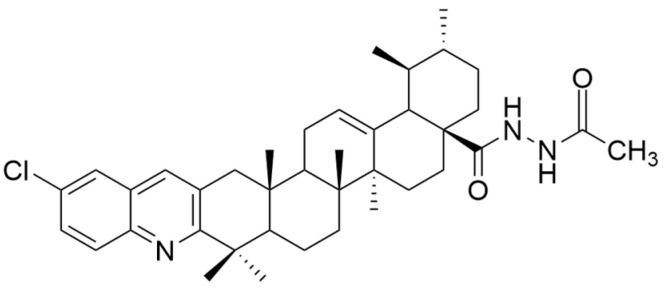
The chemical structure of compound **VII**. The structure was designed with ChemDraw 23.0.1.

Jin et al. synthesized and tested several new quinoline derivatives of UA with hydrazide, oxadiazole, or thiadiazole moieties against MDA-MB-231, HeLa, and SMMC-7721 cancer cell lines. They observed that among all the quinoline substituents, the Cl atom caused the greatest increase in cytotoxic activity. Regarding the substituents at C-28, the order of cytotoxic potency was as follows: hydrazide > carboxyl group > oxadiazole > thiadiazole [57]. Furthermore, compound **VII** (N-[5′-chloro-ursa-12-en- (2,3)-quinolin-28-oyl]-acetohydrazide) (Figure 3) demonstrated the strongest antiproliferative effect on all tested cell lines, being involved in the induction of apoptosis, increased oxidative stress, and decreased mitochondrial membrane potential [57]. UA derivatives containing an acyl piperazine moiety at C-28 prevent the proliferation of MGC-803 and Bcap-37 cancer cell lines, promoting cell apoptosis [22,71]. Several UA analogs containing a quinoline moiety show increased efficacy against MDA-MB-231, Hela, and SMMC-7721 cell lines [22,72]. It was also observed that the introduction of an isopropyl group at C-28 potentiates the antiproliferative effect of UA derivatives [66].

Da Silva et al. demonstrated that the transformation of the C3-OH into an amino group, together with the simultaneous administration of imatinib, led to a significant increase in the anticancer effect against leukemia cells [1,59]. Among all the tested compounds, a derivative containing C3-NH_2_ as a participant in the hydrogen bond network proved to be the most effective on the tested cell line, being, at the same time, harmless on healthy cells [59]. A study performed on four cancer lines (HL-60, HeLa, BGC, and Bel-7402) demonstrated that 3β-amino derivatives of ursolic acid showed an anticancer effect 20 times stronger than 3α-amino derivatives. It emphasized the importance of the configuration at C-3 for the potency of the antiproliferative effect [17,73]. Another one showed that the introduction of a 3,4,5-methoxy benzoic acid moiety at the C-3 position led to a significant cytotoxic effect against A549, MCF7, H1975, and BGC823 cancer cell lines [1,74].

**Figure 4:**
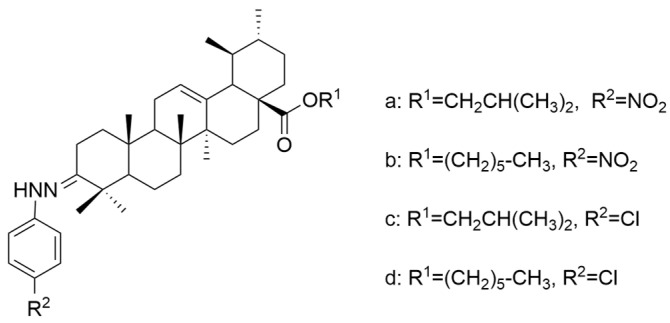
The chemical structure of compounds **X** **a**–**d**. The structures were designed with ChemDraw 23.0.1.

Meng et al. synthesized and tested 18 UA derivatives (with structural changes at C-3 and C-28) against BEL740 and SGC7901 cancer cell lines. The results showed that the most promising compounds were **Xa** and **Xc,** respectively **Xb** and **Xd** (Figure 4). Compounds **Xa** and **Xc** presented a 4′-nitro-phenylhydrazone radical at C-3, while compounds **Xb** and **Xd** presented a 4′-chloridephenylhydrazone radical in the same position. Regarding the ester group in the C-28 position, the most beneficial alkyl side chains have been shown to be isobutyl (**Xa** and **Xc**) and hexyl (**Xb** and **Xd**), respectively [60]. This study underlined that the C-3 oxidation and the alkyl side chain length strongly influence these compounds’ anticancer activity [60]. Moreover, Li et al. compared the anticancer efficacy of UA and its derivative **XI** against breast cancer cell lines SUM149PT and HCC1937. The results indicated that the new derivative presented a superior antiproliferative and proapoptotic effect compared to UA, which suggests that the introduction of a piperazine residue in C-28 and a fused pyrazole at C-3 are beneficial for increasing efficacy [61].

**Figure 5:**
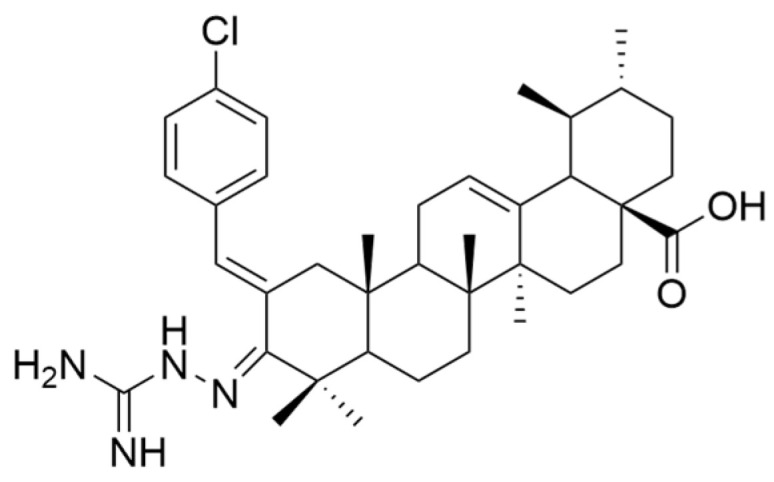
The chemical structure of compound **XIII**. The structure was designed with ChemDraw 23.0.1.

Wu and his collaborators obtained three novel series of UA derivatives by replacing the -OH group in C-3 with an aminoguanidine moiety. Testing the compounds obtained (including compound **XIII**) (Figure 5) on the cancerous cell line Hep3B, they observed that the presence of a methyl group at C-28 increases the anticancer potential, while the extension of the hydrocarbon chain causes opposite effects [63].

**Figure 6:**
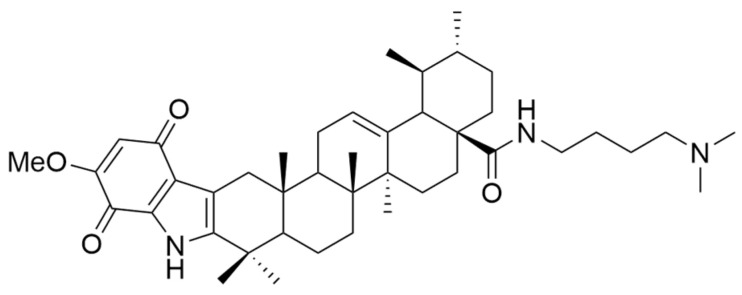
The chemical structure of compound **XIV**. The structure was designed with ChemDraw 23.0.1.

Furthermore, Tian et al. aimed to obtain new compounds derived from OA and UA by introducing various substituents at the C-28 level and testing them against three malignant cell lines (MCF-7, Hela, and A549 cell lines) [75]. The results showed that UA derivatives that had primary amines in their structure presented a more pronounced antiproliferative activity than compounds with secondary or tertiary amines [4,75]. In the study conducted by Wang et al., a series of new indolequinone derivatives of ursolic acid was obtained and tested against malignant cell lines MCF-7, HeLa, and HepG2. Following the experiments, it was observed that the introduction of indoquinoline moiety increased the anticancer potential of UA derivatives. Moreover, some derivatives having an N-(dimethylamino) alkyl moiety at the C-28 amide side chain (**XIV**) (Figure 6) demonstrated higher cytotoxic potential than other derivatives [64].

**Figure 7:**
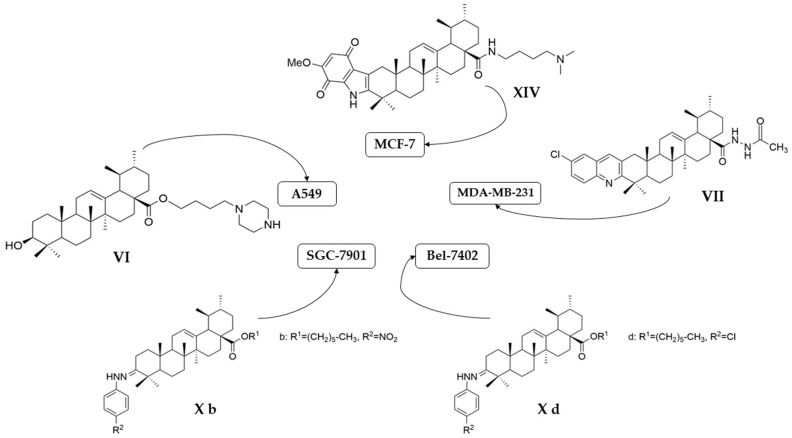
Chemical structures of the most potent antiproliferative derivatives on the selected cell lines. The structures were designed with ChemDraw 23.0.1.

Following IC_50_ value evaluation, some remarks regarding the antiproliferative activity of the discussed UA derivatives against the most tested cell lines can be made. It can be observed that compound **VII** exhibited the strongest antiproliferative effect on the MDA-MB-231 cell line (IC_50_ = 0.12 μM after 72 h following stimulation). Further, the proliferation of A549 cells was strongly inhibited by compound **VI** (IC_50_ = 5.4 μM after 72 h following stimulation), and the proliferation of MCF-7 cells was influenced by compound **XIV** (IC_50_ = 1.66 μM after 72 h following stimulation). Bel-7402 cell line proliferation was intensively affected by **Xd** analog (IC_50_ = 4.49 μM after 48 h following stimulation), while **Xb** analog induced the strongest antiproliferative effect on the SGC-7901 cell line (IC_50_ = 6.30 μM after 48 h following stimulation). The mentioned derivatives are presented in Figure 7.

Other researchers aimed to develop new UA hybrids in order to obtain improved anticancer properties of UA. Thus, Sun et al. synthesized and tested 16 ursolic acid/glycyrrhetinic acid–uracil/thymine hybrids on A549 and HeLa cancer cell lines. The results of this study suggest that glycyrrhetinic acid hybrids show antiproliferative effects slightly superior to those of UA [43]. Looking from another perspective, Isakovic and collaborators conducted an in vitro study evaluating the antiproliferative effect of betulinic acid (BA), OA, UA, and the OA+UA combination on the human metastatic melanoma cell line WM-266-4 [8]. After 48 h of incubation, the OA+UA combination (3.5:1 and 1:1) presented the highest inhibition rate, suggesting that the association between these two related compounds can lead to a superior effect [8].

It has been shown that UA inhibits tumor growth in several in vivo studies, for example, in an orthotopic colorectal nude mouse model, leukemic nude mouse model, postmenopausal breast cancer mouse model, and others (Table 3) [13,27]. Some of the most representative studies are listed below.

The study conducted by Zhang et al. has proven that UA prevents mouse S180 tumor proliferation [77]. Moreover, Wang et al. showed that UA significantly suppressed tumor growth and the occurrence of metastases in both the zebrafish and mouse xenotransplantation models of breast cancer. This result is of high importance since UA was not observed to cause nephro-, hepato-, or hematotoxicity [68]. It has also been shown that UA reduces the density of blood microvessels in murine models of colorectal cancer due to the inhibition of some key factors in the angiogenesis process (VEGF-A and bFGF) [76]. Several preclinical tests using human xenograft models have shown that UA and its derivatives have good therapeutic and chemopreventive properties [13].

An extensive study conducted by Lin et al. followed the activity of UA in vitro (human colon carcinoma cell line HT-29), in vivo (CRC mouse xenograft model), and in ovo (chicken chorioallantoic membrane) [76]. The obtained data showed that UA inhibits tumor growth, apparently without signs of toxicity, due to the inhibition of tumor angiogenesis [76]. In addition, using human umbilical vein endothelial cells (HUVECs), this study showed that the angiogenic effect of UA is dependent on dose and/or time, implying the interaction with several factors of angiogenesis [76].

In the specialized literature, there are few data comparing the anticancer effect of UA with the standard anticancer treatment. A study by Sołtys et al. involved the use of doxorubicin as a reference compound in the evaluation of the antiproliferative activity of UA and some UA derivatives on several cancer cell lines [78]. On the other hand, there are more studies in which the synergistic effect of UA and standard anticancer substances (e.g., gemcitabine, oxaliplatin) is evaluated [79,80].

There are few clinical data in the literature regarding the safety of repeated administration and the recommended doses of UA. Only a few studies have analyzed UA liposome’s pharmacokinetic profile and tolerability [13,37,38]. Therefore, further clinical studies are needed.

## 3. Oleanolic Acid

OA (3β)-3-hydroxy-olean-12-en-28-oic acid) has the basic oleanane skeleton. OA is found in more than 1600 plant species such as *Panax ginseng* C.A.Mey., *Panax pseudoginseng* Wall., *Syzygium aromaticum* L., *Glycyrrhiza glabra* L., *Lantana camara* L., *Lisgustrum lucidum* W.T.Aiton, *Gentiana lutea* L., and *Vitex doniana* Sweet, but it is particularly found in the *Oleaceae* family [10,81,82,83,84,85]. It can be easily procured from food products included in the normal diet, such as various fruits, virgin olive oil, or red wine [86,87]. Among the plant products that contain significant amounts of OA, apple skin (0.96 mg/dry weight (DW)), peach skin (1.49 mg/dry skin), pear skin (1.25 mg/dry skin), bilberries whole fruit (1679–2030 μg/g DW), grapes peel (176.2 μg/g dry weight), and olives skin (3094–4356 μg/g fresh weight (FW)) can be listed [81]. OA can be extracted from various plant products using organic solvents such as methanol, ethanol, ethyl acetate, acetone, or 1-butanol [88]. Furthermore, DMSO and DMF are used to solubilize OA in various aqueous buffers [88]. The classic extraction methods include maceration, heat reflux, or Soxhlet, while among the modern methods are ultrasonification-assisted extraction, microwave-assisted extraction, pressurized liquid extraction, and supercritical fluid extraction [23,88].

OA has been used clinically for the treatment of hepatitis [89]. It is included in one of the most widely used Chinese herbal formulas used worldwide (Rehmannia Six Formula) [90]. Furthermore, it has been marketed and used for decades as an over-the-counter hepatoprotective drug [91]. It has gained special attention due to its multiple beneficial properties, such as anti-inflammatory, hepatoprotective, neuroprotective, cardioprotective, renoprotective, antioxidant, anti-aging, immunomodulatory, anti-osteoporosis, and diuretic activities, showing great therapeutic potential [81,85,86,89,91,92,93,94]. Moreover, OA has a vast antiviral and antimicrobial effect, being active against the *Human immunodeficiency virus*, *Hepatitis C virus*, *Influenza virus*, *Herpes simplex virus*, some species of *Plasmodium*, *Mycobacterium tuberculosis*, and many pathogenic bacteria (*Staphylococcus aureus*, *Bacillus subtilis*, *Enterococcus faecium*, *Pseudomonas aeruginosa*) [83,92,95]. OA has a useful prophylactic and curative role in various pathologies, such as dyslipidemia, diabetes, metabolic syndrome, multiple sclerosis, hepatitis, and ulcerative colitis [81,87,88]. OA and its derivatives have already been successfully tested in various types of malignancies (gastric, breast, colorectal, liver, prostate, pancreatic, gallbladder, ovarian, endometrial, lung, melanoma, retinoblastoma) [8,10,82,86,89,92,96,97,98]. Recently, it was found that OA and its derivatives can also prevent cancer occurrence [89].

OA is well-known for its antioxidant potential. It is believed that the only phenolic hydroxyl group of OA is involved in the free radical scavenging activity [99]. In addition to the direct free radical scavenging mechanism, OA also presents an indirect, biological mechanism that improves the body’s antioxidant defenses, which is much more important than the direct one [99]. In their study, Sasikumar et al. analyzed the antioxidant and antiproliferative capacity of OA extracted from *Vitis vinifera* L. fruits (black raisins). It was stated that OA possessed a DPPH scavenging activity (88.3%) similar to standard antioxidant compounds such as ascorbic acid, gallic acid, pyrogallol, or butylated hydroxytoluene. Moreover, within the 3-(4,5-dimethylthiazol-2-yl)-2,5-diphenyl-tetrazolium bromide) MTT test, OA demonstrated that it is effective against HCT-116 cells, presenting an IC_50_ value of 40 μg/mL after 48 h of incubation [86]. It was also demonstrated that OA extracted from *Vitis labrusca* B. peel exhibited good antioxidant properties. The value obtained in the FRAP assay (0.77 ± 0.08 FRAP value) was slightly higher than that of gallic acid. Additionally, OA showed a lipid peroxidation inhibition rate of 24.66%, as well as a DPPH radical scavenging effect of 85.3% [100]. Gao and colleagues have tested the antioxidant activity of OA extracted from *Ligustrum lucidum* W.T.Aiton on alloxan-induced diabetic rats. They noticed a decrease in MDA activity and an increase in SOD and GPx activity, respectively [23].

As in the case of UA, the therapeutic use, including the oral administration of OA, is reduced due to its very poor water solubility (1.748 μg/L), absorption, biomembrane permeability, and low bioavailability [84,88]. Therefore, in an attempt to improve these properties, new dosage forms of OA were prepared (nanoparticles, liposomes, solid dispersions, and phospholipid complexes), and new derivatives of OA were synthesized [101].

### 3.1. Structural Modifications

OA is also a compound with limited hydrophilicity. To obtain semi-synthetic compounds with superior pharmacokinetic, dynamic, and toxicological properties, various structural modifications of OA were made. Table 4 includes the main compounds with antiproliferative effects proven in various in vitro, in vivo, and clinical studies. The structures were designed with ChemDraw 23.0.1.

It was stated that in order for OA derivatives to have antitumor properties, the functional groups at C-3 and C-28 are essential [109]. Among the most common are the transformation of the A, C, and E rings, modification of C3-OH, and transformation of C17-COOH to esters or amides [33]. The stereochemistry of the C3-OH group has special implications for the therapeutic activity of the compound [33]. Among various changes made at C3, some have been proven to improve the anticancer effect of OA. It is known that 3-oxooleanolic acid exerts a significant anticancer effect in vivo against many cancer types, especially melanoma [110]. It has been shown that the cytotoxic activity of OA against PC-3, A549, and MCF-7 cell lines can be increased by substituting the hydrogen-bond acceptor from C-3 [111]. Another point of interest for studies aimed at obtaining OA derivatives is the C-28 position. It has been proven that the amidation of C17-COOH is pharmaceutically superior to esterification, and among the amides, the morpholides and imidazolides have proven to be the most effective [26].

**Figure 8:**
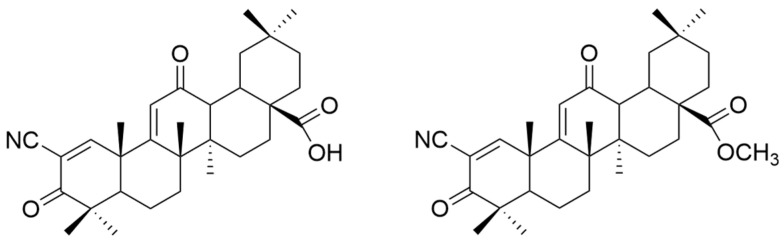
The chemical structures of compounds **XXV** and **XXVI**. The structures were designed with ChemDraw 23.0.1.

It is mentioned that a series of C-17 heteroaryl derivatives of OA have antioxidant potential [33]. Other OA derivatives such as **XXV** (2-cyano-3,12-dioxooleana-1,9(11)-dien-28-oic acid or bardoxolone) and **XXVI** (bardoxolone methyl) (Figure 8) also possess antioxidant activity. Even in nanomolar concentration, they cause an increase in the transcriptional activity of nuclear factor (erythroid-derived 2)-like 2 (Nrf2), which is a regulator of the cellular antioxidant response [112]. Even if their antioxidant mechanism is not fully known, it is believed to be related to the suppression of inductible nitric oxide synthase (iNOS) in the cells responsible for innate immunity and the reduction of the expression of TNF-α, certain interleukins (IL-1β, IL-6), and γ-interferon in various cells [112].

**Figure 9:**
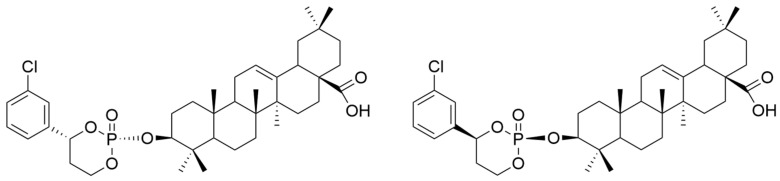
The chemical structures of the new prodrugs of **OA**. The structures were designed with ChemDraw 23.0.1.

Yu et al. synthesized two new prodrugs of OA (cis-3-O-[4-(R)-(3-chlorophenyl)-2-oxo-1,3,2-dioxaphosphorinan-2-yl]-oleanolic acid and cis-3-O-[4-(S)-(3-chlorophenyl)-2-oxo-1,3,2-dioxaphosphorinan-2-yl]-oleanolic acid) (Figure 9), then analyzed their hepatoprotective capacity against carbon tetrachloride (CCl_4_)-induced liver injury in mice. The obtained data showed that OA derivatives caused a decrease in serum transaminases and MDA and an increase in the level of enzymes with an antioxidant role (GPx and SOD), respectively. These observations indicate that these two compounds have antioxidant and hepatoprotective potential [113].

### 3.2. In Vitro, In Vivo, and Clinical Trials

Many studies showed that OA and its derivatives are effective against various cancer cell lines (thyroid, ovarian, pancreatic, breast, colorectal, lung, gallbladder, gastric cancer, glioma, and leukemia) and tried to explain the anticancer mechanism (Table 5) [10,14,26,82].

Numerous in vitro studies have been attempted to demonstrate the antiproliferative effect of OA. Some of the representative ones that tried to explain the anticancer mechanism of this compound are briefly described below.

Nie et al. observed that OA is effective against multiple human gastric cancer cell lines (SGC-7901, MGC-803, and BGC-823) since it induces autophagic death [89]. Using the SW579 thyroid cancer cell line, Duan et al. showed that OA inhibits the proliferation and induces apoptosis of cancer cells by targeting forkhead transcription factor A [82]. Woo et al. showed that OA is effective against A375SM and A375P melanoma cells, with the proapoptotic effect being mediated by the NF-κB pathway [115]. Another study on SMMC-7721 human hepatocellular carcinoma showed that cell apoptosis induced by OA is closely related to the alteration of mitochondrial function [116].

**Figure 10:**
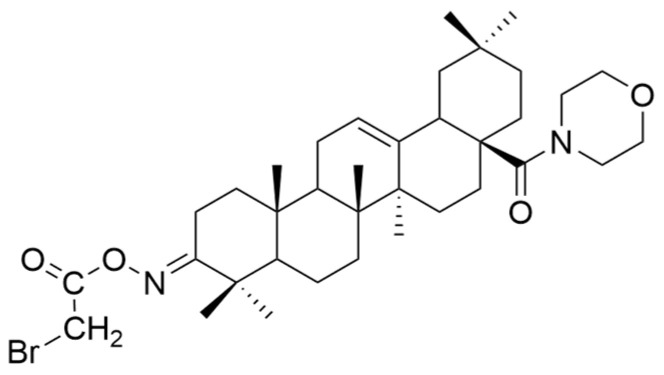
The chemical structure of compound **XV b**. The structure was designed with ChemDraw 23.0.1.

Also, some OA derivatives have been proven to be effective in vitro. Some of these studies are listed below, emphasizing the structural changes that led to improving the anticancer effect. The role of pyrimidine as an antitumor pharmacophore has been extensively used in several experiments that aimed to obtain compounds with superior anticancer properties [90]. Meng and his collaborators demonstrated that the antitumor activity of OA derivatives against SGC-7901 and A-549 cell lines increases with the increase in ester chains. Additionally, they suggested that the introduction of a quinoxaline ring to ring A of OA determines the improvement of the anticancer properties of the derivatives [109]. Bednarczyk et al. synthesized four new OA derivatives and tested them against human melanoma cell lines MeWo and A375. Among these compounds, **XV b** (Figure 10), which was a bromoacetoxyimine derivative, showed the best activity on the tested cell lines [26]. This study demonstrated that alkyl derivatives are preferred over aryl ones [26].

Various attempts considered the simultaneous modification of C-3 and C-28, obtaining satisfactory results. Fontana et al. synthesized and tested several derivatives of OA and UA against hepatocellular carcinoma cell lines HepG2, Hep3B, and HA22T/VGH. They observed that the various modifications at the C-3 and C-28 positions determined changes in the anticancer potential. More specifically, acetylation of the C3-OH group is unfavorable in both cases, whereas methylation of the C17-COOH does not significantly influence the activity of UA derivatives, but it may be favorable, unfavorable, or irrelevant for some OA derivatives [62].

**Figure 11:**
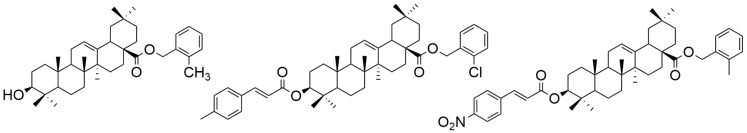
The chemical structures of compounds **XVIII**, **XIX**, and **XX**. The structures were designed with ChemDraw 23.0.1.

On a different line, Sun and his collaborators applied pharmacophore hybridization to obtain new compounds with improved anticancer effects. They prepared several oleanolic acid–uracil/thymine conjugates and demonstrated, within the MTT test, that these hybrids possess superior pharmacological activity to oleanolic acid, respectively 5-fluorouracil [43]. Thus, obtaining conjugated compounds can contribute to increasing the therapeutic effect and widening the spectrum of anticancer compounds [43]. Mo et al. considered synthesizing and evaluating the antiproliferative effect of acyl oleanolic acid–uracil conjugates. They have proven that the compound with a propionyloxy group at C-3 was the most effective against Hep-G2, while the compound with a dodecanoyloxy group at C-3 was the most effective against A549. Furthermore, the most effective compound against MCF-7 had an acetoxy group at C-3. Regarding the PC-3 cell line, the best results were obtained with the butyryloxy compound. This study concludes that, in general, the acylation of the C3-OH group potentiates the antiproliferative activity of OA [90]. It is already known that, besides OA, cinnamic acid (CA) is a compound that exhibits anticancer properties. This was the starting point of the study led by Wang, which aimed to synthesize new OA-CA derivatives by using a molecular hybridization approach and test them against HeLa (cervical cancer) and MCF-7 (breast cancer) [102]. Among these new derivatives, none proved to be effective on both cell lines, but three of them (**XVIII**, **XIX**, and **XX**) (Figure 11) exerted a strong antiproliferative effect on a malignant cell line, suggesting that further research in this area is needed.

**Figure 12:**
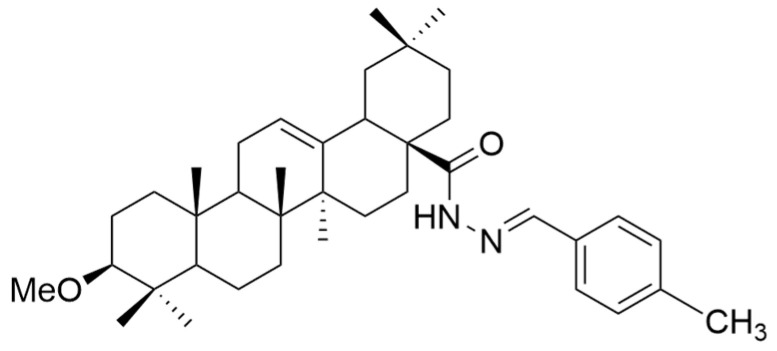
The chemical structures of compound XXII a. The structure was designed with ChemDraw 23.0.1.

**Figure 13:**
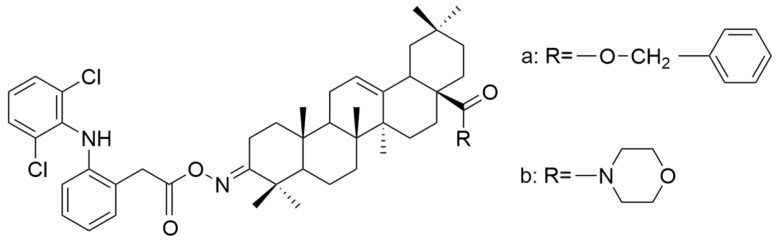
The chemical structures of compounds **XXI a** and **XXI b**. The structures were designed with ChemDraw 23.0.1.

Several oleanolic acid hydrazide–hydrazone hybrids were synthesized and tested on the A549 human lung cancer cell line by Halil et al. The structure of OA was modified as follows: the C3-OH was methylated to methyl ether, and the C17-COOH group was transformed into a hydrazide. Then, the hybrids were synthesized starting from the obtained hydrazide and another 13 different aromatic aldehydes [104]. Among the tested hybrids, 4-methylbenzaldehyde hydrazone (**XXII a**) (Figure 12) showed the best results, having cytotoxic properties equivalent to doxorubicin against cancer cells and being 32 times less toxic to healthy cells [104]. Narozna et al. analyzed the antiproliferative effect of diclofenac (DCL)-OAO conjugates against the HepG2 liver cancer cell line. The obtained results showed that the conjugation of diclofenac with OA derivatives with a morpholide group or benzyl ester in the C-28 position (**XXI a** and **XXI b**) (Figure 13) leads to an increase in the anticancer action [103].

**Figure 14:**
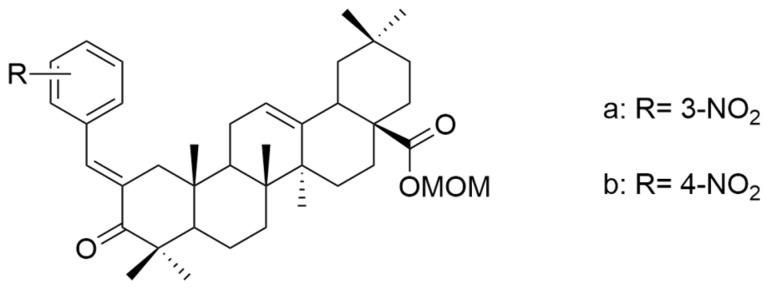
The chemical structures of compounds **XXIII a** and **XXIII b**. The structures were designed with ChemDraw 23.0.1.

In terms of ring A modifications, Şenol et al. analyzed new derivatives of α,β-unsaturated ketones based on oleanolic acid against a human prostate cancer cell line (PC3). The obtained data concluded that compounds with the nitro group at the meta- and para-positions of the phenyl ring (**XXIII a** and **XXIII b**) (Figure 14) were the most potent against cancer cells [105].

**Figure 15:**
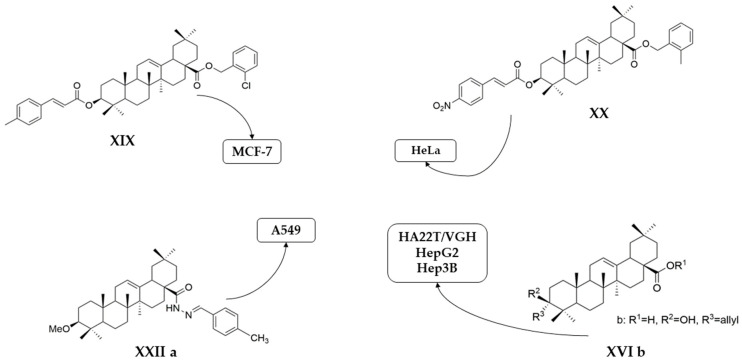
Chemical structures of the most potent antiproliferative derivatives on the selected cell lines. The structures were designed with ChemDraw 23.0.1.

In the same manner as the UA derivatives, IC_50_ values of OA derivatives were evaluated for the most tested cancer cell lines. The **XIX** derivative exhibited the strongest antiproliferative effect on the MCF-7 cell line (IC_50_ = 1.79 μM after 48 h following stimulation). A549 cells were strongly inhibited by **XXII** a compound, with an IC_50_ value of only 0.08 μM after 24 h following stimulation. **XVI b** compound showed potent antiproliferative effect against hepatocellular carcinoma HepG2, Hep3B, and HA22T/VGH cell lines (IC_50_ values being 28.0 μM, 32.5 μM, and 31.0 μM respectively, after 72 h following stimulation). An intense antiproliferative effect against HeLa cells was observed for **XX** compound (IC_50_ = 1.35 μM after 48 h following stimulation). The mentioned derivatives are presented in Figure 15.

**Figure 16:**
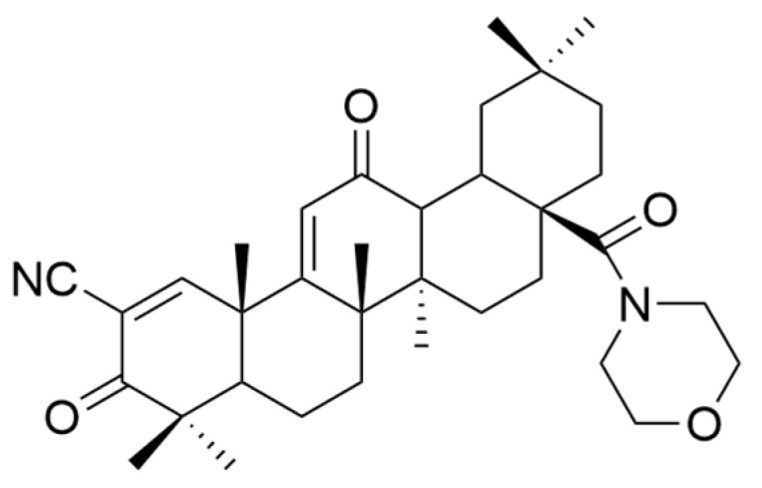
The chemical structures of compound **XXIV**. The structure was designed with ChemDraw 23.0.1.

Various attempts were made to obtain OA derivatives with superior properties. Thus, several series of compounds were discovered, including **XXV** (bardoxolone), which can be considered 200,000 times more potent than OA [33]. Compound **XXVI** (bardoxolone methyl) also demonstrated antiangiogenic and antitumor effects in rodent cancer models [90]. Of this series, compound **XXVI** is considered to be the most promising in cancer treatment [33]. Gao et al. synthesized another compound of interest, **XXIV** (N-formylmorpholine substituent of **XXV**) (Figure 16), which was tested in vitro and in vivo. They found that this compound inhibited osteosarcoma cell growth due to decreased c-MYC-dependent glycolysis [106]. This study suggests that compound **XXIV** may become a valuable antitumor compound [106].

Regarding in vivo studies, the anticancer efficacy of OA and its derivatives have been demonstrated using various experimental animal models (Table 6).

A study in tumor-bearing mice demonstrated that OA significantly reduced the mass of cervical tumors. The suggested mechanism is that of increasing oxidative stress, Fe^2+^ concentration, and the expression of ferroptosis-related proteins [117]. OA 3-acetate (**XVII**) was shown to be highly effective in reducing tumor growth on tumor xenografts with SKOV3 cells in immunocompromised mice [97].

Cheon et al. used an animal model of testosterone-induced benign prostatic hyperplasia (BPH) to test the efficacy of OA. The obtained data showed that OA determined the reduction of BHP symptoms, having a superior effect compared to finasteride [118]. Another study designed on prostate cancer xenografts in mice suggested that OA causes p53-dependent apoptosis via the ERK/JNK/AKT pathway [96].

Using a colorectal cancer mouse xenograft model, it was also demonstrated that OA causes cancer cell apoptosis by increasing the expression of BAX, P21, and p53 and by inhibiting the expression of Bcl-2, CKD-4, and Cyclin D1 [119]. Moreover, in murine models, it was observed that OA shows chemopreventive activity against 1, 2-dimethylhydrazine-induced colon carcinoma [89,120].

It was also demonstrated on nude mice transplanted with pancreatic cancer L3.6PL cells that intragastric administration of the bardoxolone methyl caused significant tumor inhibition (74.2%) [77].

The literature is poor on clinical data regarding the anticancer activity of OA and its derivatives (Table 7). In clinical trials, the results of compound **XXVI** are inconclusive. It was observed that the pharmacokinetic characteristics of compound **XXVI** include non-linearity, slow oral absorption, long half-life, and pronounced inter-individual variability. The maximum tolerated dose was 900 mg/d. Moreover, in a patient with mantle cell lymphoma, a complete tumor response was observed, while a partial response was recorded in a patient with anaplastic thyroid cancer [108]. Even if this compound appears to be well tolerated, it has been noted that it may increase the risk of cardiovascular events [121].

## 4. Conclusions

The data presented in this review highlight the anticancer potential of pentacyclic triterpenes UA and OA, mostly in preclinical studies. It also underlines the main structural changes that can improve the efficacy of these compounds (such as transformation into esters or amides, replacing the OH group with a hydroxyimine group, the introduction of a pyrimidine or quinoline moiety, hybridization with uracil, thymine, hydrazide, or cinnamic acid, etc.). Currently, there are a plethora of in vitro studies conducted on various cancer cell lines that have demonstrated the antiproliferative and cytotoxic effects of UA, OA, and their derivatives. Most of the semi-synthetic derivatives presented in this review showed a better antiproliferative effect than the natural compound against specified cancer cell lines. Moreover, some compounds displayed an antiproliferative effect comparable or superior to certain anticancer drugs on the pharmaceutical market (e.g., compounds **VII** and **XIV** to etoposide, compounds **X a-d** to adriamycin). However, in vivo studies and especially clinical trials are scarce; hence, bringing together the whole picture regarding pentacyclic triterpenes ursolic and oleanolic acids and related derivatives as anticancer candidates can help open new research avenues on this topic. Further research in this area is needed by including new semi-synthetic terpenoid derivatives in animal studies and further clinical trials.

## Figures and Tables

**Table 1:** Chemical structures of UA derivatives.

Number	Chemical Structure	Reference
I	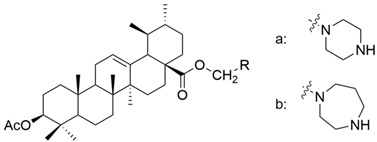	[22]
II	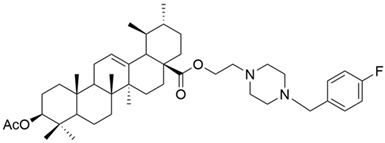	[22]
III	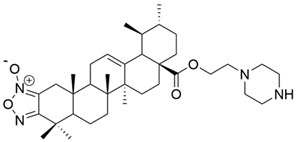	[22]
IV	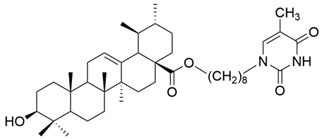	[43]
V	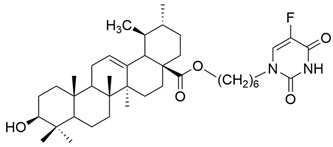	[16]
VI	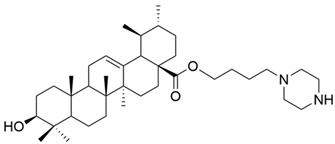	[56]
VII	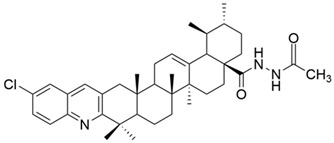	[57]
VIII	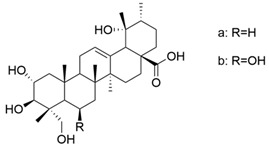	[58]
IX	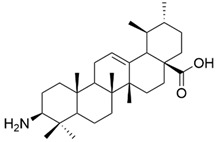	[59]
X	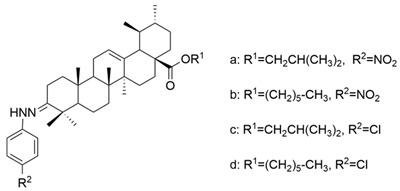	[60]
XI	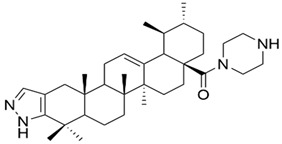	[61]
XII	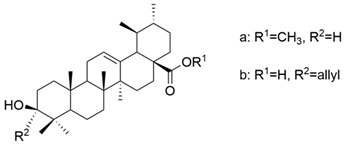	[62]
XIII	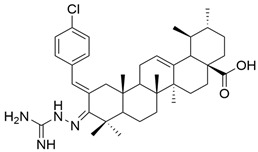	[63]
XIV	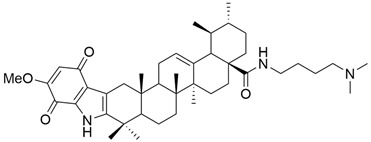	[64]

**Table 2:** In vitro studies regarding the anticancer effect of UA and its derivatives.

Compound	Type of Cancer Cell Line	Cell Line	In Vitro	Conclusions	Reference
**UA**	human leukemic monocyte lymphoma	U-937	MTT IC_50_ = 36.59 μM	HL-60 cell line was more sensitive to the antiproliferative action of UA compared to the U-937 line.	[25]
	human acute promyelocytic leukemia	HT-60	MTT IC_50_ = 26.83 μM		[25]
	human breast cancer	MDA-MB-231	Methylene blue assay (72 h) (antiproliferative activity) EC_50_ = 18.12 μM	UA significantly inhibited the proliferation of MCF-7 cells in a dose-dependent manner without being cytotoxic.	[27]
	human ovarian cancer	SKOV-3 OC	CCK8 (48 h) (Cell viability assay) IC_50_ = 35 μM	UA caused a significant decrease in the viability of SKOV-3 cells.	[28]
	non-small cell lung cancer	A549	MTT (24 h) IC_50_ ≥ 20 μM	UA inhibited cell proliferation in a dose-dependent manner.	[67]
	non-small cell lung cancer	H460	MTT (24 h) IC_50_ ≥ 20 μM		[67]
	human breast cancer	MDA-MB-231	CCK8 (48 h) IC_50_ = 24 μM	UA inhibited cell proliferation in a dose- and time-dependent manner.	[29]
		MCF-7	CCK8 (48 h) IC_50_ = 29.2 μM		[29]
			CCK8 (48 h) IC_50_ = 7.96 μM	UA significantly suppressed the proliferation of cancer cells in a doze-time-dependent manner.	[68]
		MDA-MB-231	CCK8 (48 h) IC_50_ = 9.02 μM		[68]
		MCF-7	MTT (24 h) IC_50_ = 20 μM	UA determined the decrease in the cell viability in a dose-dependent manner.	[69]
	human gingival squamous carcinoma	Ca922	MTT (48 h) IC_50_ = 11.5 μM	UA exhibited good antiproliferative effects.	[70]
	human oral squamous carcinoma	SCC2095	MTT (48 h) IC_50_ = 13.8 μM		[70]
	human breast cancer	SUM149PT	SRB (48 h) IC_50_ = 8–10 μM	UA significantly inhibited proliferation of cancer cells.	[61]
		HCC1937	SRB (48 h) IC_50_ = 8–10 μM		
**UA**	human skin metastatic melanoma	WM-266-4	MTT (4, 24, 48 h) The lowest efficient concentration = 10 μM	Both UA and UA+OA decreased cell proliferation.	[8]
**UA+OA** **(both 1:1 and 3.5:1)**					[8]
**Ia**	human gastric cancer	MKN45	MTT (72 h) IC_50_ = 6.4 μM	The inhibition rate was above 75%, which means that the introduction of a piperazine or homopiperazine radical increases the antitumor activity.	[22]
**Ib**			MTT (72 h) IC_50_ = 6.2 μM		[22]
**II**			MTT (72 h) IC_50_ = 2.1 μM		[22]
**III**			MTT (72 h) IC_50_ = 4.5 μM		[22]
**IV**	human liver cancer	HL-7702	MTT (72 h) IC_50_ = 42.1 μM	UA thymine hybrids had a slightly lower antiproliferative activity than glycyrrhetinic acid hybrids.	[43]
**V**	non-small cell lung cancer	A549,	MTT (48 h) IC_50_ = 17.35 μM	This compound presented antiproliferative activity against all cancer cell lines.	[16]
	human breast cancer	MCF-7	MTT (48 h) IC_50_ = 18.86 μM		[16]
	human hepatocellular carcinoma	Bel-7402	MTT (48 h) IC_50_ = 32.50 μM		[16]
	human myelogenous leukemia	K562	MTT (48 h) IC_50_ = 14.89 μM		[16]
**VI**	non-small cell lung cancer	A549	CCK9 (24 h) IC_50_ = 6.1 μM	The new derivative exhibited significantly better antiproliferative effect than UA.	[56]
			CCK9 (48 h) IC_50_ = 5.5 μM		
			CCK9 (72 h) IC_50_ = 5.4 μM		
		H460	CCK9 (24 h) IC_50_ = 5.7 μM		[56]
			CCK9 (48 h) IC_50_ = 4.5 μM		
			CCK9 (72 h) IC_50_ = 3.9 μM		
**VII**	human breast cancer	MDA-MB-231	MTT (72 h) IC_50_ = 0.12 μM	This acylhydrazine derivative showed the strongest antiproliferative effect against all three cancer cell lines tested, being superior to etoposide.	[57]
	cervical carcinoma	HeLa	MTT (72 h) IC_50_ = 0.08 μM		[57]
	hepatocarcinoma	SMMC-7721	MTT (72 h) IC_50_ = 0.34 μM		[57]
**VIII (a+b)**	human laryngeal carcinoma	Hep-2	MTT (48 h) IC_50_ = 6.8 μM	These derivatives presented a significant antiproliferative effect against cancer cells.	[58]
	human hypolaryngeal carcinoma	FaDu	MTT (48 h) IC_50_ = 6.78 μM		[58]
**IX**	human chronic myelogenous leukemia	K562	MTT (48 h) IC_50_ = 5.2 μM	This compound was the most effective antiproliferative agent among all tested derivatives, showing low cytotoxicity against normal cells.	[59]
**X a**	human hepatoma	BEL-7402	MTT (48 h) IC_50_ = 7.08 μM	These compounds showed antiproliferative activity that was comparable to stronger than its positive control drugs (VP-16 and adriamycin).	[60]
	human gastric cancer	SGC-7901	MTT (48 h) IC_50_ = 15.62 μM		
**X b**	human hepatoma	BEL-7402	MTT (48 h) IC_50_ = 8.57 μM		[60]
	human gastric cancer	SGC-7901	MTT (48 h) IC_50_ = 6.30 μM		
**X c**	human hepatoma	BEL-7402	MTT (48 h) IC_50_ = 5.63 μM		[60]
	human gastric cancer	SGC-7901	MTT (48 h) IC_50_ = 8.73 μM		
**X d**	human hepatoma	BEL-7402	MTT (48 h) IC_50_ = 4.49 μM		[60]
	human gastric cancer	SGC-7901	MTT (48 h) IC_50_ = 7.01 μM		
**XI**	human breast cancer	SUM149PT	SRB (48 h) IC_50_ = 4–6 μM (for both cell lines)	This compound significantly inhibited the proliferation of cancer cells, exhibiting very low toxicity on normal cells. Moreover, this compound showed better antiproliferative activity than UA.	[61]
		HCC1937			
**XII a**	human hepatocellular carcinoma	Hep3B	MTT (72 h) IC_50_ = 38.0 μM	These compounds showed a superior antiproliferative effect compared to UA against only one cell line among the three tested (HA22T/VGH, HepG2, and Hep3B)	[62]
**XII b**			MTT (72 h) IC_50_ = 40.0 μM		[62]
**XIII**			HRE (24 h) (luciferase reporter assay) IC_50_ = 4.0 μM	This compound inhibited the HIF-1α transcriptional activity, which is a very important factor in tumor growth.	[63]
**XIV**	human breast cancer	MCF-7	MTT (72 h) IC_50_ = 1.66 μM	This compound displayed a better antiproliferative effect than UA against all tested cell lines and better activity than Etoposide against MCF-7 and HeLa cell lines, with low toxicity against normal cells.	[64]
	human cervical carcinoma	HeLa	MTT (72 h) IC_50_ = 3.16 μM		
	human hepatocarcinoma	HepG2	MTT (72 h) IC_50_ = 10.35 μM		

**Table 3:** In vivo studies regarding the anticancer effect of UA and its derivatives.

Compound	Experimental Animal Model	Injected Tumor Cells	Concentration	Conclusions	Reference
**UA**	Male BALB/c athymic nude mice	human colon carcinoma (HT-29)	12.5 mg/kg, i.p, 6 days/week, 16 days	UA inhibited tumor growth without apparent toxicity.	[76]
**UA**	Chick chorioallantoic membrane (CAM)	-	10 μL of UA (25 μg/μL), 72 h of incubation	UA inhibited angiogenesis.	[76]
**UA**	Female Balb/c mice	breast cancer (4T1-Luc)	25 and 50 mg/kg/day i.p., measuring tumor volume every 3 days	UA suppressed the proliferation of cancer cells and prevented the occurrence of lung metastasis without significant body weight loss.	[68]
**UA**	Nude mouse subcutaneous xenograft model	human retinoblastoma (SO-RB50)	200 mg/kg, i.p., twice a week, 7 weeks	UA suppressed the tumor growth.	[30]
**VIII (a+b)**	Female BALB/c nude mice, 4 weeks old	human laryngeal carcinoma (Hep-2)	45 and 90 mg/kg/day, 28 days	Administration of VIII (a+b) resulted in inhibition of tumor growth without significant weight loss.	[58]

**Table 4:** Chemical structures of OA derivatives.

Number	Chemical Structure	Reference
XV	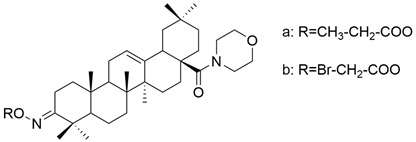	[26]
XVI	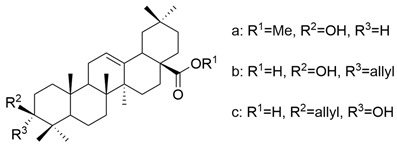	[62]
XVII	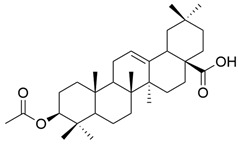	[97]
XVIII	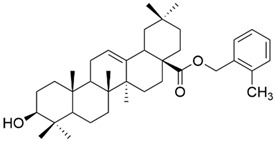	[102]
XIX	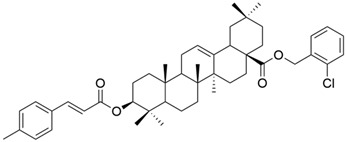	[102]
XX	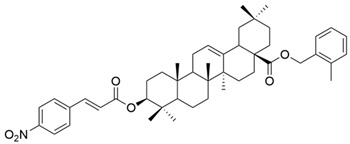	[102]
XXI	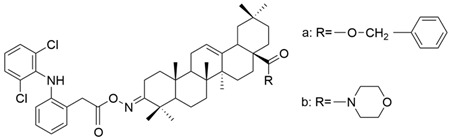	[103]
XXII	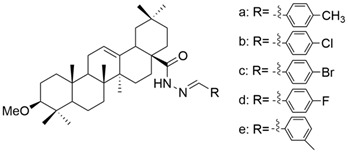	[104]
XXIII	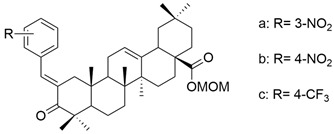	[105]
XXIV	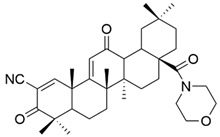	[106]
XXV	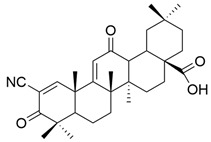	[107]
XXVI	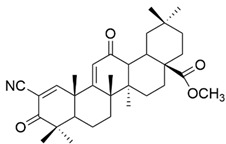	[108]
XXVII	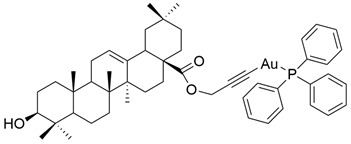	[19]
XXVIII	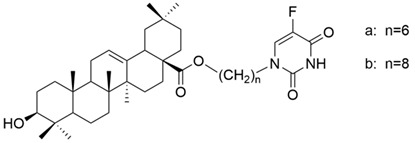	[16]

**Table 5:** In vitro studies regarding the anticancer effect of OA and its derivatives.

Compound	Type of Cancer Cell Line	Cell Line	In Vitro	Conclusions	Reference
**OA**	human skin metastatic melanoma	WM-244-6	MTT (24 h, 48 h) The lowest concentration that caused a significant antiproliferative effect was 20 μM.	OA inhibited cancer cell proliferation, showing a maximal effect after 48 h.	[8]
**OA**	human gastric cancer	SGC-7901	MTT (48 h) IC_50_ = 25.9 μM MTT (72 h) IC_50_ = 21.2 μM	OA decreased the viability of cancer cells in a dose-dependent manner, showing slight inhibition against normal cells.	[89]
		MGC-803	MTT (48 h) IC_50_ = 24.0 μM MTT (72 h) IC_50_ = 20.4 μM		
		BGC-823	MTT (48 h) IC_50_ = 30.1 μM MTT (72 h) IC_50_ = 22.4 μM		
**OA**	human breast carcinoma	MCF-7	MTT (48 h) IC_50_ = 13.09 μg/mL	OA exhibited dose-dependent antiproliferative activity against cancer cells with negligible toxicity against normal cells.	[10]
		MDA-MB-231	MTT (48 h) IC_50_ = 160.22 μg/mL		
**OA**	human colon adenocarcinoma	HCT-116	MTT (48 h) IC_50_ = 40.00 μg/mL	OA significantly decreased the cancer cells’ viability.	[86]
**OA**	human thyroid carcinoma	SW579	MTT (24 h) IC_50_ = 42.20 μmol/L	OA exhibited a dose-dependent antiproliferative effect against cancer cells.	[82]
**OA**	liver carcinoma	HepG2	CCK (24 h) IC_50_ = 30 μM	OA determined a dose-dependent decrease in cancer cells’ viability without significant toxicity against normal cells.	[114]
**OA**	human melanoma	A375SM	MTT (24 h) The 100 μM dose reduced the cell viability to 41.5%.	OA decreased the cell viability in a dose-dependent manner.	[115]
		A375P	MTT (24 h) The 100 μM dose reduced the cell viability to 46.4%.		[115]
**OA**	human prostate cancer	DU145	MTT (24 h) IC_50_ = 112.57 μg/mL	OA inhibited the proliferation of cancer cells.	[96]
	human breast cancer	MCF-7	MTT (24 h) IC_50_ = 132.29 μg/mL		
	human glioblastoma	U87	MTT (24 h) IC_50_ = 163.6 μg/mL		
**OA**	human melanoma	MeWo	MTT (48 h) OA did not have a significant antiproliferative effect against MeWo cells.	Derivatives XVa and XVb exhibited dose-dependent antiproliferative effects against both cancer cell lines, being superior to OA.	[26]
		A375	MTT (48 h) The 100 μM dose reduced the cell viability to 61%.		
**XV a**		MeWo	MTT (48 h) The 100 μM dose reduced the cell viability to 37.2%.		[26]
		A375	MTT (48 h) The 100 μM dose reduced the cell viability to 16.4%.		
**XV b**		MeWo	MTT (48 h) The 100 μM dose reduced the cell viability to 10.9%.		[26]
		A375	MTT (48 h) The 100 μM dose reduced the cell viability to 16%.		
**XVI a**	hepatocellular carcinoma	HA22T/VGH	MTT (72 h) IC_50_ = 42.5 μM	These compounds showed a superior antiproliferative effect compared to OA against all tested cell lines.	[62]
		HepG2	MTT (72 h) IC_50_ = 40.0 μM		
		Hep3B	MTT (72 h) IC_50_ = 41.5 μM		
**XVI b**		HA22T/VGH	MTT (72 h) IC_50_ = 31.0 μM		[62]
		HepG2	MTT (72 h) IC_50_ = 28.0 μM		
		Hep3B	MTT (72 h) IC_50_ = 32.5 μM		
**XVI c**		HA22T/VGH	MTT (72 h) IC_50_ = 35.8 μM		[62]
		HepG2	MTT (72 h) IC_50_ = 44.0 μM		
		Hep3B	MTT (72 h) IC_50_ = 33.7 μM		
**XVII**	human ovarian cancer	SKOV3	MTT (24 h) IC_50_ = 8.3 μM	This compound exhibited strong antiproliferative activity against cancer cells without significant toxicity against normal cells.	[97]
	human endometrial cancer	HEC-1A	MTT (24 h) IC_50_ = 0.8 μM		[97]
**XVIII**	human cervical cancer	HeLa	MTT (48 h) IC_50_ = 1.55 μM	Compounds XVIII and XX exhibited strong antiproliferative activity against HeLa cells. Compound XIX was the most efficient against MCF-7 cells.	[102]
	human breast cancer	MCF-7	MTT (48 h) IC_50_ = 32.49 μM		
**XIX**	human cervical cancer	HeLa	MTT (48 h) IC_50_ > 100 μM		[102]
	human breast cancer	MCF-7	MTT (48 h) IC_50_ = 1.79 μM		
**XX**	human cervical cancer	HeLa	MTT (48 h) IC_50_ = 1.35 μM		[102]
	human breast cancer	MCF-7	MTT (48 h) IC_50_ > 100 μM		
**XXI a**	human hepatoma	HepG2	MTT (24 h) IC_50_ = 37.0 μM	These compounds presented better antiproliferative activity than diclofenac.	[103]
**XXI b**			MTT (24 h) IC_50_ = 33.5 μM		[103]
**XXII a**	human non-small lung cancer	A549	MTT (24 h) IC_50_ = 0.08 μM	Compound XXII a had the greatest cytotoxic activity (equivalent to doxorubicin) against cancer cells while being less toxic to normal cells.	[104]
**XXII b**			MTT (24 h) IC_50_ = 0.35 μM		[104]
**XXII c**			MTT (24 h) IC_50_ = 0.31 μM		[104]
**XXII d**			MTT (24 h) IC_50_ = 1.72 μM		[104]
**XXII e**			MTT (24 h) IC_50_ = 0.22 μM		[104]
**XXIII a**	human prostate cancer	PC3	MTT (24 h) IC_50_ = 7.79 μM	All of these compounds presented good antiproliferative effects against cancer cells, being superior to OA.	[105]
**XXIII b**			MTT (24 h) IC_50_ = 8.87 μM		[105]
**XXIII c**			MTT (24 h) IC_50_ = 8.77 μM		[105]
**XXVII**	human ovarian cancer	A2780	MTT (72 h) IC_50_ = 10.24 μM	This gold alkyne complex was more active than OA against cancer cells.	[19]
**XXVIII a**	non-small cell lung cancer	A549	MTT (48 h) IC_50_ = 23.44 μM	The obtained hybrids possess antiproliferative properties. The hybrid XXVIII b presented potential selectivity against cancer cells and moderate antiproliferative potential against both ordinary and multidrug-resistant (MDR) A549/T and Bel-7402/FU cell lines.	[16]
	human breast cancer	MCF-7	MTT (48 h) IC_50_ = 24.33 μM		[16]
	human hepatocellular carcinoma	Bel-7402	MTT (48 h) IC_50_ = 25.22 μM		[16]
	human myelogenous leukemia	K562	MTT (48 h) IC_50_ = 14.92 μM		[16]
**XXVIII b**	non-small cell lung cancer	A549,	MTT (48 h) IC_50_ = 50.54 μM		[16]
		A549/T	MTT (48 h) IC_50_ = 43.07 μM		[16]
	human breast cancer	MCF-7,	MTT (48 h) IC_50_ = 53.63 μM		[16]
		MCF-7/ADR	MTT (48 h) IC_50_ = 166.2 μM		[16]
	human hepatocellular carcinoma	Bel-7402	MTT (48 h) IC_50_ = 43.82 μM		[16]
		Bel-7402/FU	MTT (48 h) IC_50_ = 31.42 μM		[16]
	human myelogenous leukemia	K562	MTT (48 h) IC_50_ = 22.99 μM		[16]
		K562/ADR	MTT (48 h) IC_50_ > 200 μM		[16]

**Table 6:** In vivo studies regarding the anticancer effect of OA and its derivatives.

Compound	Experimental Animal Model	Injected Tumor Cells	Concentration	Conclusions	Reference
**OA**	Female nude BALB/c mice, 6 weeks old	human gastric cancer (MGC-803)	100 mg/kg/day, orally, measuring the tumor volume every 3 days	OA inhibited tumor growth and delayed the onset of tumor formation.	[89]
**OA**	Female BALB/c nude mice, 4 weeks old	human melanoma (A375SM)	75 mg/kg and 150 mg/kg, i.p., 5 times/week, 13 days	OA (150 mg/kg dose) caused a significant reduction in the tumor volume.	[115]
**OA**	Male BALB/c nude mice, 5 weeks old	human cervical cancer (Hela)	40 and 80 mg/kg/day, i.p., 15 days	OA decreased the size of cervical cancer tumors.	[117]
**OA**	Male Sprague-Dawley rats, 8 weeks old	human benign prostate hyperplasia (BHP-1)	1 and 10 mg/kg/day, i.p., 4 weeks	OA treatment determined the reduction of prostate tissue weight (by 30.91%, and 31.23%) without affecting the body weight.	[118]
**OA**	Male BALB/c mice, 7 weeks old	human prostate cancer (DU145)	50 μg/mouse, i.m., every 2 days, 4 times	OA significantly decreased the net weight of tumors (about 29.38%).	[96]
**XVII**	Female athymic mice, BALB/c nu/nu, 6–8 weeks old	human ovarian cancer (SKOV3)	10, 20, and 40 mg/kg/day, i.p., 3 weeks	XVII (OA-3 acetate) exhibited significant tumor growth inhibition rates (32.5, 38.47, and 46.02%) without causing weight loss.	[97]
**XXIV**	Female BALB/c nude mice, 5 weeks old	human osteosarcoma (143B)	40 mg/kg, i.p., every 2 days, 3 weeks	This compound reduced the tumor volume without affecting the body weight and other organs.	[106]
**XXVII**	Male BALB/c nude mice	human ovarian cancer (A2780)	20 mg/kg/day, i.p., 15 days	This complex determined a significant tumor inhibition rate (40.2%) without clear weight loss.	[19]

**Table 7:** Clinical trials.

Compound	Clinical Trial Phase/Type	Subjects	Type of Cancer	Dose	Results	Conclusions	Reference
**XXV**	open-label, single-arm phase I study	two males, five females, aged 47–62	colorectal (4), bladder (1), ovarian (1), and uterine (1) cancer	Seven different dose levels (0.6 to 38.4 mg/m^2^/h) in continuous infusion on days 1–5 of a 28-day cycle	Bardoxolone pharmacokinetics showed no evidence of non-linearity. The desired dose level of 1 µM was reached only after the administration of the highest dose.	The study was discontinued due to the occurrence of thromboembolic events.	[107]
**XXVI**	phase I	47 patients (34 males and 13 females), aged 24–81	solid tumors or refractory lymphoid malignancies	5 mg/d (starting dose) administrated orally for 21 days of a 28-day cycle	The dose-dependent toxicity consists of a reversible increase in ALT serum level.	Compound XXVI was a well-tolerated drug. Thus, the dose of 900 mg/d could be recommended for phase II clinical trials.	[108]

## Data Availability

All the extracted data are presented in the manuscript.
